# Elevated Proangiogenic Markers are Associated with Vascular Complications within Ghanaian Sickle Cell Disease Patients

**DOI:** 10.3390/medsci6030053

**Published:** 2018-06-27

**Authors:** Charles Antwi-Boasiako, Emmanuel Frimpong, Ben Gyan, Eric Kyei-Baafour, Fredericka Sey, Bartholomew Dzudzor, Mubarak Abdul-Rahman, Gifty B. Dankwah, Kate H. Otu, Tom A. Ndanu, Andrew D. Campbell, Ivy Ekem, Eric S. Donkor

**Affiliations:** 1 School of Biomedical and Allied Health Sciences, University of Ghana, Accra, Ghana; dzudzorb@yahoo.com (B.D.); presmubarak@gmail.com (M.A.-R.); gdankwah@gmail.com (G.B.D.); ericsdon@hotmail.com (E.S.D.); 2 School of Medical Sciences, College of Health and Allied Sciences, University of Cape Coast, Cape Coast, Ghana; frimemma@gmail.com (E.F.); ekem_ivy@hotmail.com (I.E.); 3 Noguchi Memorial Institute for Medical Research, University of Ghana, Accra, Ghana; BGyan@noguchi.ug.edu.gh (B.G.); Ekyei-baafour@noguchi.ug.edu.gh (E.K.-B.); 4 Sickle Cell Clinic, Korle-Bu Teaching Hospital, Accra, Ghana; fredisey@hotmail.com; 5 Department of Nursing and Midwifery, Greenhills School of Health Sciences, Accra, Ghana; kateboasiako@yahoo.co.uk; 6 School of Medicine and Dentistry, University of Ghana, Accra, Ghana; nutcaresoft@gmail.com; 7 Center for Cancer and Blood Disorder, Hematology Children’s National Medical Center, George Washington School of Medicine and Health Sciences, Washington, DC 20052, USA; acampbell@childrensnational.org

**Keywords:** sickle cell disease, angiogenic factors, angiopoietin-1, angiopoietin-2, vascular endothelial growth factor

## Abstract

Sickle cell disease (SCD) is an inherited blood disorder that can result in vasculopathy and end organ damage. Angiogenesis has been implicated as a key contributing factor to vascular mediated tissue injury in SCD. The relative plasma levels of angiopoietin-1 (Ang-1), angiopoietin-2 (Ang-2), and vascular endothelial growth factor (VEGF) greatly influence angiogenesis. Dysregulation of these growth factors, leading to a pro-angiogenic state in SCD patients, has been documented in the developed world but there is very little data in Africa. There is the need, therefore, for studies in Ghanaian SCD patients. The aim of this study was to assess plasma levels of Ang-1, Ang-2, and VEGF in homozygous (HbSS) SCD patients with or without complications and healthy controls (HbAA) in Ghana. The study was a case-control study involving 544 participants: 396 HbSS SCD patients and 148 HbAA healthy controls. The study was conducted at the Center for Clinical Genetics (Sickle Cell Clinic) and Accra Area Blood Centre for National Blood transfusion at the Korle-Bu Teaching Hospital, Accra, Ghana. The plasma levels of Ang-1, Ang-2, and VEGF of study participants were measured with a double sandwich enzyme-linked immunosorbent assay (ELISA) technique. Complete blood count (CBC) was measured with an autoanalyser. The mean plasma Ang-1, Ang-2, and VEGF were significantly higher in HbSS SCD patients with or without complications than healthy controls (*p* < 0.001). The Ang-2/Ang-1 ratio was significantly lower in the controls than the HbSS patients (*p* < 0.001). The Ang-2/Ang-1 ratio was higher in the HbSS patients with leg ulcers as compared with patients with other complications and healthy controls (*p* < 0.001). There were higher leucocyte counts in HbSS patients than healthy controls. Overall, there was elevated plasma levels of Ang-1, Ang-2, and VEGF in SCD patients. The higher Ang-2/Ang-1 plasma levels in patients with leg ulcers suggests a possible ongoing angiogenesis and response to inflammatory stimuli. The study provides a first report on plasma levels of angiopoietin-1, angiopoietin-2, and vascular endothelial growth factors in homozygous sickle cell disease patients in Ghana.

## 1. Introduction

In sickle cell disease (SCD), the repeatedly sickled red blood cells eventually fail to return to normal shape when normal oxygen tension is restored, referred to as irreversible sickle cells [1]. As a result, these rigid blood cells are unable to deform as they pass through the capillaries, leading to vessel occlusion and ischemia [1]. This causes poor microvascular blood flow, with consequent tissue ischemia, hypoxia, and infarction [1,2]*,* and can further result in several complications in SCD patients, including vaso-occlusive crises (VOC), leg ulcers, priapism, acute chest syndrome, kidney failure, pulmonary hypertension, osteonecrosis and retinopathy, cerebro-vascular accident, and splenic infarction with repeated bacterial infection [2,3].

Vascular occlusion is the hallmark of vaso-occlusive crises in SCD [4]. The micro-vascular occlusion, with subsequent ischaemia-reperfusion injury, may result in vasculopathy [5]. Subsequently, the vascular lesion due to VOC-induced tissue hypoxia may lead to a potent angiogenic response [6]. These major physiological stimuli for angiogenesis are all sequelae of acute sickle cell VOC, including tissue ischaemia, hypoxia, inflammation, and shear stress [7]. Moreover, vascular growth can be stimulated by ischaemia and hypoxia alone [8]. Thus, considering frequent vascular occlusions, inflammation, leukocytosis, and reperfusion tissue injury [2], angiogenesis to various degrees may be inevitable in SCD patients. Leg ulcers are the most frequent cutaneous manifestation of SCD, which may contribute to morbidity, with an incidence of 25% to 75% among affected patients [9,10]. Priapism is one of the less well characterized complications of the SCD disorder [11], with a prevalence of 2–6% in paediatric sickle-cell clinics [12].

Angiogenesis is a highly regulated process and requires coordinated signalling events among a variety of angiogenic factors [13]. Angiogenic growth factors, such as angiopoietin-1 (Ang-1), angiopoietin-2 (Ang-2), and vascular endothelial growth factor (VEGF), play crucial roles in developmental blood vessel formation and regulation of hypoxia-induced tissue angiogenesis [14], among other angiogenic growth factors. Vascular endothelial growth factor has been established as the prime angiogenic molecule during organogenesis, as well as post-natal physiological and pathological angiogenesis [14]. It is the most potent stimulator of endothelial cell proliferation, sprouting, migration, and tube formation, as well as a powerful survival factor and permeability factor for endothelial cells [15]. Angiopoietins are involved in the early processes of angiogenesis and vasculogenesis [16]. There are four identified angiopoietins, namely: Ang-1, Ang-2, Ang-3, and Ang-4, with two corresponding tyrosine kinase receptors: Tie-1 and Tie-2 [17]. Ang-1 and Ang-2 are specific ligands of Tie-2 [17]; however, Ang-1 is a major agonist to the Tie-2 receptor [18]. In healthy individuals, plasma levels of Ang-1 exceed that of Ang-2 [18]. Binding of Ang-1 to Tie-2 promotes vessel integrity, inhibits vascular leakage, and suppresses inflammatory gene expression [17]. Ang-1 acts in a paracrine agonistic manner, inducing Tie-2 phosphorylation and subsequent vessel stabilization [17]. Thus, Ang-1 facilitates endothelial cell sprouting and vascular network maturation [19].

On the other hand, Ang-2 is exclusively expressed by endothelial cells and only found at sites of tissue re-modelling [18]. Ang-2 acts as an autocrine antagonist of Ang-1-mediated Tie-2 activation [17] and promotes apoptosis, as well as vessel regression in the absence of VEGF [20], but facilitates endothelial cell migration and proliferation with VEGF [20,21]. Therefore, high Ang-2 relative to Ang-1 and VEGF levels favour angiogenesis and responsiveness to hypoxic and inflammatory stimuli [22].

While plasma or serum levels of these angiogenic factors have been extensively studied in other disease populations, such as coronary artery disease, hypertension, sepsis, pulmonary hypertension, and diabetes mellitus, as measures of vasculopathy, angiogenesis, and disease mortality [23,24,25], few studies have been conducted on plasma levels of these angiogenic factors in SCD patients. Prior to this study, there were no previous studies on the relative levels of Ang-1, Ang-2, and VEGF in SCD patients with a haemolytic clinical sub-phenotype, including leg ulcers and priapism, in Ghana, where 2% of all births in Ghana are born with SCD or Sub-Saharan Africa, although reports indicate that angiogenic progenitors increase in VEGF in homozygous (HbSS) patients [6,23,26]. The current study aimed to investigate the relative plasma levels of Ang-1, Ang-2, and VEGF among SCD patients in Ghana.

## 2. Methods

### 2.1. Study Setting and Study Population

The study design was a case-control study. The study was conducted at the Korle Bu Teaching Hospital, Accra, Ghana from February 2013 to July 2014. The cases (SCD patients) and controls were recruited from the Centre for Clinical Genetics (Sickle Cell Clinic) and Accra Area Blood Centre for National Blood Transfusion, respectively. The patients were recruited on site, after their routine hospital visitation and consultation. The healthy controls were recruited at the Accra Area Blood Centre for National Blood Transfusion after medical examination. The study involved 544 participants, including 396 HbSS SCD patients and 148 HbAA healthy controls. Out of the 396 HbSS patients, 208 were in steady state (without complication), 156 in VOC, 21 with leg ulcers, and 11 with priapism. The study protocol was approved by the Ethical and Protocol Review Committee of the University of Ghana Medical School. All the study participants gave written informed consent after the study procedures were thoroughly explained to them. Ethical Approval for the study was obtained from the Ethical and Protocol Review Committee of University of Ghana Medical School (Protocol identification number + MS-Et/M.11–P 5.7/2012-2013).

Steady state was clinically defined as a patient who has been well and has not been in crisis for at least two weeks [27]. Vaso-occlusive crisis was clinically defined as pains in the bones, muscles, and joints not attributable to any other cause, requiring parenteral analgesia, and admitted in the Sickle Cell Centre [28]. A leg ulcer was defined as a defect in the skin below the level of the knee and above the foot, persisting for six or more weeks [29]. Sickle cell disease patients with leg ulcers recruited in the current study were not necessarily active. Priapism was defined as a purposeless, persistent, penile erection not accompanied by sexual desire or stimulation, lasting more than six hours [30]. However, patients with conditions which affect plasma Ang-1, Ang-2, and VEGF levels, such as diabetes mellitus, hypertension, coronary artery disease, renal failure, pregnancy, and recent blood transfusion three months prior to the study, were excluded from the study [31].

### 2.2. Blood Sampling and Laboratory Measurements

Five millilitres (5 mL) of venous blood sample was collected from each of the study participants into ethylene diamine tetracetic acid (EDTA) tubes. An aliquot of 2.5 mL of the blood sample was processed into plasma and stored at −80 °C. A complete blood count (CBC) was done with 2.5 mL of the blood sample within 2 h of collection using labsystemmultiskan MS (Amisham Bioscience LTD, Little Chalfont, UK).

### 2.3. Assays of Angiopoietin-1, Angiopoietin-2, and Vascular Endothelial Growth Factor

The plasma levels of Angiopoietin-1 and Angiopoietin-2 of HbSS SCD patients and controls were measured by a double sandwich ELISA technique (R&D DuoSet ELISA Development kit) according to the manufacturer’s instructions. The optical density was determined within 30 min using a microplate reader set to 450 nm (Amersham Bioscience Limited, Little Chalfont, UK).

The plasma levels of VEGF were measured by a double sandwich ELISA technique (R&D DuoSet ELISA Development kit, Minneapolis, USA) according to the manufacturer’s instructions. The optical density was determined within 30 min, using a microplate reader set to 450 nm (EL808 BioTekmicroplate, Loughborough, UK).

### 2.4. Data Analysis

The data was entered in SPSS version-20 software. Frequency tables were generated for nominal and ordinal variables. The results are expressed as the mean plus or minus standard deviation (mean ± SD). The Kruskal Wallis test was used to compare differences in mean values among SCD patients in steady state and VOC, as well as those with leg ulcers and priapism, with the healthy controls. A Dunn’s test was done as a post hoc analysis for multiple comparisons. Statistical significance was considered at *p* < 0.05.

## 3. Results

### 3.1. Study Participants

The 544 enrolled subjects included 148 HbAA healthy controls (93 males, 55 females) and 396 HbSS SCD patients (199 males, 197 females). While a predominance of males was represented within the control group (63%), there was equivocal gender representation within the HbSS subject group (50%). The mean age of the HbAA controls was 31.9 years (± 10.0) and 25.5 (± 9.7) years for the HbSS SCD group. Details regarding the demographics, gender, and medical history of the subjects are described in Table 1. Sixty percent were in steady state (*n* = 208), 45% (*n* = 156) were in active VOC, 5% (*n* = 21) had an active leg ulcer, and 3% (*n* = 11) had an active priapism. Males had twice as many leg ulcers (*n* = 14) than females (*n* = 7) in symptomatic patients. However, females (*n* = 92) comprised of most VOC (92 vs 64 males) patients.

### 3.2. Complete Blood Count of Participants

The mean plasma levels of haemoglobin (Hb), haematocrit (HCT) were significantly lower in HbSS patients with or without complications than the HbAA controls (*p* < 0.001). Also, mean platelet count (PLT), and white blood cells (WBC) were significantly higher in HbSS patients with or without complications than HbAA (*p* < 0.001) (Table 1).

### 3.3. Mean Plasma Angiopoietin-1, Angiopoietin-2, and VEGF Levels in Healthy HbAA Controls, Steady State HbSS Patients, and HbSS Patients with Complications (Vaso-Occlusive Crisis, Leg Ulcers, and Priapism)

The mean plasma Ang-1 was significantly higher in HbSS patients with complications (VOC, leg ulcers, and priapism) or without complications (steady state) compared to the healthy controls (*p* < 0.001) (Table 2). The relative plasma levels of these angiogenic factors is recorded in Table 2.

Plasma Ang-2 was significantly higher in HbSS patients with complications or without complications than the healthy controls (p < 0.001). Plasma VEGF was significantly higher in HbSS patients with complications and without complications than the healthy controls (*p* < 0.001). Similar to Ang-1 and Ang-2, plasma VEGF in HbSS patients with complications was higher than HbSS patients in steady state.

The Ang-2/Ang-1 ratios of HbSS patients in steady state, HbSS patients with complications, and healthy HbAA controls were also analysed. Ang-2/Ang-1 ratio was higher in the HbSS patients with complications, especially leg ulcer patients, compared to the healthy controls (*p* < 0.001) (Table 3).

## 4. Discussion

Several angiogenic growth factors, including Ang-1, Ang-2, and VEGF, have been found to be elevated in SCD patients generally [26]. Angiogenesis is a physiological process involving the growth of new blood vessels or neo-vascularization [32]. This is a vital process for embryological growth, tissue development, and wound healing in damaged tissues [32]. In this study, we found that the mean plasma Ang-1, Ang-2, and VEGF levels were significantly elevated in SCD patients as compared to the controls. By comparison, previous studies conducted elsewhere found higher levels of these angiogenic growth factors in SCD patients [6,23,26,33]. While the majority of previous studies focused on SCD patients generally, the current study enrolled patients with haemolytic clinical sub-phenotypes, including leg ulcers and priapism, and determined their relative levels of some angiogenic growth factors associated with angiogenesis in plasma.

The rise in plasma levels of these angiogenic growth factors could partly lead to angiogenesis. Although higher levels of VEGF were recorded in clinically asymptomatic SCD patients compared to controls in some studies [23,34], the study conducted by Duits et al. [6] found lower levels among SCD patients with the HbSS hemoglobin. However, in the clinically asymptomatic state, patients’ Ang-2 level was elevated when compared with controls. Thus, the increase in one angiogenic growth factor does not necessarily lead to the elevation of other angiogenic factors to promote or support angiogenesis. In line with our study, Mohan et al. [29] reported elevated Ang-2, VEGF, and Ang-1 plasma levels in clinically asymptomatic SCD patients [30]. Although these levels were elevated in the asymptomatic SCD patients, those with complications recorded higher levels. Meanwhile, a similar study reported that angiogenesis is influenced by (and not necessarily caused) the relative levels of Ang-1, Ang-2, and VEGF [31]. High Ang-2 relative to Ang-1 and VEGF levels favoured angiogenesis and responsiveness to hypoxic, as well as inflammatory, stimuli in neoplasms [22]. Thus, the high level of Ang-2/Ang-1 ratio observed in the SCD patients with leg ulcers suggests a possible increased angiogenesis. Increased systemic tissue hypoxia usually observed in SCD patients (as well as those with a haemolytic clinical sub-phenotype) may have contributed to the elevated plasma Ang-1, Ang-2, and VEGF levels recorded in this study [16].

In the current study, higher levels of the angiogenic factors (Ang-2, VEGF, and Ang-1) recorded in SCD patients with vaso-occlusion is worth noting. Vaso-occlusion is the hallmark of SCD [4], with elevated white blood cells [35]. Ang-2 and soluble vascular cell adhesion molecule (sVCAM) levels have been found to be high in painful crises of SCD [6]. The associated pain that comes with vaso-occlusion in SCD patients may have, in part, influenced the observed elevated levels of the angiogenic growth factors, as well as, possibly, the higher white blood cells in these SCD patients. A possible link between vaso-occlusion and elevated white blood cells has been reported in a study conducted elsewhere [26]. Therefore, elevated white blood cells, as well as an increase in angiogenic growth factors, could possibly serve as risk factors for the development of severe complications of SCD [36]. Ang-1 has been shown to have anti-apoptotic effects on endothelial cells. Thus, perhaps, an ongoing similar endothelial damage in SCD patients with leg ulcers and priapism may have contributed, in part, to the similar (elevated) levels of Ang-1 for provision of support [37,38]. The difference in levels of these angiogenic factors observed in all complications associated with the SCD patients studied could be because of variations in the ongoing vasculopathy. Vaso-occlusion development in SCD is usually associated with inflammation and vasomotor dysregulation [6]. Thus, SCD severity and sub-phenotypes could possibly influence levels of angiogenic factors, as seen in the current study. While the study conducted by Duits et al. [6] reported elevated plasma levels of Ang-1 and VEGF among HbSS patients with painful crises and those in the asymptomatic state, respectively, the current study, however, found that all plasma levels of both Ang-1 and VEGF, as well as Ang-2, further increased during crises and decreased during the steady state.

Whether angiogenic growth factors are markers of sickle cell disease severity seems unclear [6]. The current finding did not establish a correlation between the three angiogenic growth factors and sickle cell disease severity. We, however, reported that the angiogenic growth factors studied were lower in SCD patients in the steady state, as well as their healthy counterparts. The need to determine a possible association and correlation between angiogenic growth factors and disease severity of SCD is crucial, as this would help clinicians in the management of SCD and associated complications. Other studies have used serum, instead of plasma, to quantify levels of these angiogenic growth factors elsewhere [6]. However, in line with our study, another study used plasma to quantify levels of Ang-1, Ang-2, and VEGF and reported higher levels [30]. It is intriguing to have seen elevated levels of haemoglobin in patients with VOC, compared with those in the asymptomatic state in this study. In line with this finding, a previous study conducted in Nigeria reported higher haemoglobin levels in HbSS VOC, compared with those at the steady state, although the difference was not significant [29].

The main limitation of this study was the inability to measure other anti-angiogenic markers (such as thrombospondin, VEGF165b, angiostatin, endostatin), making the interpretation of the circulating angiogenic factors difficult, since these levels may also influence angiogenesis. The cases recruited in the current study were higher than the controls. Nevertheless, the finding of this study from the Ghanaian community could serve as a basis for further work in other complications of SCD in the same population or other sub-Saharan countries. We predict, based on our findings, that the levels of these angiogenic growth factors may increase further in other acute complications of SCD and that the majority of adults (of mean age ≥ 25 years) may suffer from vascular injury.

In normal vascular physiology, there is a balance between inhibitors and angiogenic growth factors that regulate angiogenesis. However, the problem in SCD patients is the predisposition and experience of repeated vaso-occlusion that results in ischaemic organ damage. When the regulatory mechanisms of angiogenesis fail, blood vessels are either formed excessively or insufficiently [39], which may result in further SCD complications.

## 5. Conclusions

The study provides a first report on plasma levels of angiopoietin-1, angiopoietin-2, and vascular endothelial growth factors, as well as Ang-2/Ang-1 ratios, in homozygous sickle cell disease patients in Ghana. These factors were generally high in SCD and further elevated in patients with complications. Additionally, patients with VOC, a complication of SCD, recorded higher plasma levels of Ang-1, Ang-2, and VEGF. The study, indeed, cannot infer causality from the design, although there were higher concentrations of angiogenic markers in some complications of the SCD herein studied. All the same, the current study has shown that SCD patients with HbSS VOC in Ghana (sub-Saharan Africa) may experience a possible angiogenesis. It is important to mention that higher levels of only angiogenic markers in study participants is not evidence of angiogenesis. Further studies could be carried out to validate the current findings among other complications of SCD.

## Figures and Tables

**Table 1:** Demographics, clinical history, and complete blood cell count (CBC) within homozygous sickle haemoglobin (HbSS) study participants.

All Category	Control	HbSS Steady	HbSS with Complications (*n* = 189)		
Total *N* = 544	HbAA 27.2% (*n* = 148)	State 38.2% (*n* = 208)	VOC * 28.7% (*n* = 156)	Leg Ulcer * 3.9% (*n* = 21)	Priapism * 2.0% (*n* = 11)
Mean age (years ± SD)	(31.9 ± 10.0)	(25 ± 9.7)	(26.2 ± 9.4)	(27.9 ± 5.6)	(30.9 ± 13.0)
Gender (Male) (Female)	62.8% (*n* = 93) 37.2% (*n* = 55)	52.9% (*n* = 110) 47.1% (*n* = 98)	41.0% (*n* = 64) 59.0% (*n* = 92)	66.7% (*n* = 14) 33.3% (*n* = 7)	100% (*n* = 11) 0% (*n* = 0)
CBC					
WBC (10^3^/mm^3^)	5.6 ± 1.2	12.2 ± 3.6	16.8 ± 7.4 *p* < 0.001 *	13.0 ± 4.8 *p* < 0.001 *	11.7 ± 3.1 *p* < 0.001 *
Hb (g/dL)	14.3 ± 3.5	8.5 ± 1.6	9.2 ± 1.7 *p* < 0.001 *	9.0 ± 1.9 *p* < 0.001 *	8.8 ± 2.1 *p* < 0.001 *
HCT (%)	41.6 ± 3.7	25.4 ± 4.5	27.6 ± 5.1 *p* < 0.001 *	27.0 ± 3.6 *p* < 0.001 *	27.1 ± 8.1 *p* < 0.001 *
PLT (10^3^/mm^3^)	237.5 ± 31.0	452.2 ± 120.4	286.6 ± 13.3 *p* < 0.001 *	238.6 ± 109.9 *p* < 0.001 *	443.0 ± 136.1 *p* < 0.001 *

HbSS—Homozygous sickle haemoglobin; VOC—vaso-occlusive crisis; *n*—sample size; mean ± SD—mean plus or minus standard deviation; CBC—Complete Blood Count; WBC—white blood cells; Hb—hemoglobin; HCT—haematocrit; PLT—platelets count; *—*p* value = HbSS Steady State vs HbSS with Complications.

**Table 2:** Comparing mean plasma angiopoietin-1 (Ang-1), angiopoietin-2 (Ang-2), and vascular endothelial growth factor (VEGF) of controls and HbSS patients in steady state, vaso-occlusive crisis (VOC), leg ulcers, and priapism.

Angiogenic Factors (pg/mL)	HbAA Control (*n* = 148) (Mean ± SD)	HbSS Steady State * (*n* = 208) (Mean ± SD)	HbSS with Complications			
			HbSS VOC ** (*n* = 156) (Mean ± SD)	HbSS Leg Ulcer ** (*n* = 21) (Mean ± SD)	HbSS Priapism ** (*n* = 11) (Mean ± SD)	*p*-Value
Ang-1	9140 ± 781 a	10,569 ± 1207 b	22,696 ± 5787 c	15,665 ±6849 d	15,598 ± 1829 e	<0.001 *<0.001 **
Ang-2	843 ± 189 a	2187 ± 1830 b	6058 ± 2525 c	5368 ± 1893 d	4219 ± 1123 e	<0.001 *<0.001 **
VEGF	37 ± 9 a	45 ± 6 b	71 ± 23 c	62 ± 11.3 d	55 ± 9 e	<0.001 *<0.001 **

*n*—sample size; mean ± SD—mean plus or minus standard deviation; HbAA— Healthy controls; Ang-1—angiopoietin-1; Ang-2—angiopoietin-2; VEGF—vascular endothelial growth factor; ^a,b,c,d,e^—Statistically significant after Dunn’s test for multiple comparisons. * p-value = HbAA Controls vs HbSS Steady State, ** *p*-value = HbSS Steady State vs HgbSS Complications (group).

**Table 3:** Comparing Ang-2/Ang-1 ratios of controls and HbSS patients in steady state, VOC, leg ulcers, and priapism.

Ang-2/Ang-1 Ratio	HbAA Control (*n* = 148)	HbSS * Steady State (*n* = 208)	HbSS with Complications			
			HbSS ** VOC (*n* = 156)	HbSS ** Leg Ulcer (*n* = 21)	HbSS ** Priapism (*n* = 11)	*p*-value
Ang-2/ Ang-1 ratio	0.09 ^a^ (1:11.1)	0.21 ^b^ (1:4.8)	0.30 ^c^ (1:3.3)	0.35 ^d^ (1:2.9)	0.27 ^e^ (1:3.7)	<0.001 *

*n*—sample size; Ang-2/Ang-1—angiopoietin-2/angiopoietin-1 ratio; * *p*-value—HbAA Controls vs HbSS Steady State, ** *p*-value—HbSS Steady State vs HgbSS with Complications (Group). Statistical Analysis of variance (ANOVA). ^a,b,c,d,e^ are all statistically significant with each other after a Bonferroni test for multiple comparisons*.*

## Availability of Data and Materials

The datasets on which the conclusions of the manuscripts rely on are available at the Department of Physiology, College of Health Sciences, and the University of Ghana. It is situated in the Korle-Bu Teaching Hospital, Accra, Ghana. The Department has the data base of all investigations done within the review period.
